# (GTG)_5_-PCR fingerprinting of multi-drug resistant *Escherichia coli* bacteria isolates from hospital in Ouagadougou, Burkina Faso

**DOI:** 10.1186/s12866-022-02537-7

**Published:** 2022-04-29

**Authors:** Boukaré Kaboré, Ganamé Abasse Ouédraogo, Hama Cissé, Henri S. Ouédraogo, Emmanuel Sampo, Koudbi Jacob Zongo, Boukaré Zeba, Yves Traoré, Olivier Gnankiné, Idrissa Sanou, Aly Savadogo

**Affiliations:** 1 Department of Biochemistry and Microbiology, Laboratory of Applied Biochemistry and Immunology, University Joseph KI-ZERBO, BP 7021 Ouagadougou 03, Burkina Faso; 2 SPS of Schiphra, Ouagadougou, Burkina Faso; 3 Department of Biochemistry and Microbiology, Faculty of Applied Science and Technology, University of Dedougou, BP 176 Dedougou, Burkina Faso; 4 Department of Biology and Physiology Aminale, University Joseph KI-ZERBO, BP 7021 Ouagadougou 03, Burkina Faso; 5 UFR Health Sciences, University Joseph KI-ZERBO, BP 7021 Ouagadougou 03, Burkina Faso; 6 Laboratory of Bacteriology and Virology at, Tengadogo University Hospital, BP 104 Ouagadougou, Burkina Faso

**Keywords:** *E. coli*, Multidrug resistance, (GTG)_5_-PCR, Molecular typing

## Abstract

**Background:**

*Escherichia coli* (*E. coli*) is the most common bacterial species implicated in various types of infections including septicemia, gastroenteritis, urinary tract infections, meningitis and others pathologies. These involve several bacterial clones with multidrug resistance making them difficult to treat. The aims of this study was to perform molecular typing of *E. coli* strains using universal primer (GTG)_5_. In this study, 53 *E. coli* strains were collected from inpatients and outpatients. The test of antimicrobial sensibility was realized using CA-SFM /EUCAST method and strains were identified by conventional microbiological tests. The carbapenemase-producing strains were demonstrated by phenotypic method. Bacterial DNA was extracted by boiling method. (GTG)_5_-PCR was used for strain subtyping. The DendroUPGMA software was used for grouping of strains from the genetic fingerprints obtained by (GTG)_5_-PCR.

**Results:**

Antibiotic susceptibility test revealed that all strains were multi-drug resistant (MDR). Its strains showed resistance to at least three different families of antibiotics. Of this MDR strains, only one was a metallo-β-lactamase producer. The dendrogram obtained using genetic fingerprinting allowed the *E. coli* strains to be grouped into 22 clusters (G1 to G22).

**Conclusion:**

The (GTG) 5-PCR assay enabled rapid molecular typing of *E. coli* strains. The strains of *E. coli* typed in this study would belong to different clones.

**Supplementary Information:**

The online version contains supplementary material available at 10.1186/s12866-022-02537-7.

## Introduction

In the clinical infectious disease research, Gram-negative bacilli are the most clinically important [1] and therefore the most studied bacteria. These bacteria produce the most formidable enzymes whose carbapenemases with either serine-based or zinc-facilitated hydrolysis mechanisms, posing some of the most critical problems [2]. *E. coli*, *Klebsiella pneumoniae*, *Acinetobacter baumannii*, and *Pseudomonas aeruginosa* are responsible for serious multi-drug resistant infections. Antibiotics resistance (AMR) is a serious threat to global public health. The problems associated with carbapenemase production are clinical in that they compromise the activity of last-resort antibiotics used to treat serious infections. They are epidemiological and are widespread in almost all regions in the world [3]. Gram-negative bacteria are the most common culprits in nosocomial diseases, particularly urinary tract infections and bloodstream infections. However, their accurate identification is necessary for better management of these infections. Some identification techniques commonly used in health centers do not allow accurate identification. In the last decade, the introduction of automated identification instruments has improved reliability, but discrepancies between different technological methods have been reported [4]. Studies have shown differences in identification results for non-fermentative gram-negative bacilli that correspond to species not included in the API-20NE, VITEK 2 databases [5]. However, there are molecular identification methods using different primers such as 16S rRNA identification [6], palindromic repetitive extragenic PCR techniques (rep-PCR) [7]. Indeed, studies have shown that the (GTG)_5_ rep-PCR technique provides good strain discrimination compared to other techniques [8]. Although this technique has been widely used to study diversity within prokaryotic microorganisms, the genetic fingerprinting obtained by rep-PCR can also be applied to microbial ecology and microbial evolution studies [9]. The objective of this study was to identify *E. coli* strains isolated from pathological products using rep-PCR fingerprinting with the universal (GTG)_5_ primer.

## Materials and methods

### Isolation and biochemical identification of bacterial strains

Strains were isolated from the pathological products of hospitalized or non-hospitalized patients. These products consisted mainly of urine and pus. These products were inoculated on culture media (CLED, EMB), which made it possible to obtain the strains. These strains were purified, pure cultures of *E. coli* were obtained by the transfer of a single colony using two successive streak plates. Pure colonies were identified using the API 20E gallery. Bacteria isolated were preserved on Luria Bertani medium (at 4 °C) and added 20% glycerol stocks (at—20 °C) for further analysis [8, 10].

### Antimicrobial susceptibility testing

Antibiotic resistance was achieved by the disk diffusion method according to the recommendations of CA-SFM/EUCAST “European Committee on Antimicrobial Susceptibility Testing” (2020). A 0.5 McFarland bacterial suspension was prepared from each isolate in saline solution and spread on Mueller–Hinton agar. After incubation strains were classified as sensitive, intermediate and resistant using the critical inhibition diameter limits (CA-SFM/EUCAST, 2020).

### Carbapenemase detection

After, carbapenem are used for detection of class B and A carbapenemase and temocillin for class D carbapenem resistant. Strains were classified susceptible, intermediary and resistant using breakpoint specified by CA-SFM/EUCAST [11]. EDTA is used on carbapenem to detect metallo-beta-lactamase [12, 13]. Boric acid in the presence of imipenem is used for the detection of class A carbapenemase and temocillin for the detection of class D carbapenemase [14].

### Molecular characterization by (GTG)_5_-PCR

#### DNA extraction

Bacterial DNA was extracted from pure cultures of *E. coli* strains using the heat shock method with 24 h bacterial cultures [15–17]. The presence of DNA was assessed by qualitative analysis in agarose gel electrophoresis. The supernatant was stored at -20° C for performing PCR [18].

#### (GTG)_5_ primers and PCR conditions

Rep-PCR was performed with the universal (GTG)_5_ primer (5'-GTGGTGGTGGTGGTG-3'). Amplification was carried out in 25 µL of reaction mixture containing 4 μL of Master Mix (FIREPol®), 1 μL of (GTG)_5_, 17 μL of nuclease-free water and 3 μL of DNA extract. Amplification consisted for 30 PCR cycles in a thermocycler (Mastercycler nexus gradient). The programm PCR was as follows: initial denaturation at 94 °C for 4 min followed by 30 cycles of denaturation at 95 °C for 30 s, annealing at 45 °C for 1 min and elongation at 65 °C for 8 min. The PCR ended with a final extension at 65 °C for 16 min, and the amplified product cooled at 4 °C [8, 19]. *E. coli ATCC 25922*, *Klebsiella pneumoniae ATCC 700603* and *Pseudomonas aeruginosa ATCC 27853* strains were used for standardization of rep-PCR reactions, to assess reproducibility and as a positive control.

#### Electrophoresis of PCR products

A volume of ten microliters of each PCR product was placed in the wells of the agarose gel. The gel was prepared from 1.5 g of agarose by adding to 4 μL of ethidium bromide (1 g/mL) in TAE buffer at 1X (pH8). Band sizes were compared using a 100 bp molecular weight marker. The migration was carried out for two hours at 60 V and 40 mA. The gel was visualized and photographed by the UVP PhotoDoc-lt Imaging System. A visual observation of the profiles obtained was made. Strains with identical profiles (characteristic bands) were assigned to the same group.

### Phylogenetic affiliation of strains

The phylogenetic affiliation of the strains was carried out thanks to the genetic fingerprints obtained by rep-PCR. IBM SPSS Statistics Software Version 25. 2017 was used for data processing. The construction of the phylogenetic tree was carried out by performing a hierarchical classification analysis by the program DendroUPGMA (Unweighted Pair Group Method with Arithmetic). The band profiles for each isolate were converted to a binary matrix, indicating the presence or absence of a characteristic band. The similarity matrix was calculated using the Jaccard coefficient index. The UPGMA algorithm was applied to the similarity matrix with at and above mean Jaccard coefficient (standard deviation) value of 88%.

## Results and discussion

### Antimicrobial susceptibility

Fifty-three MDR-type *E. coli* strains were isolated and identified from pathological products included in this study. Of these strains, 92.4% (*n* = 49) were from urinary tract infections and 7.6% (*n* = 4) from pus. Indeed, we retain as MDR in this study, any strain of *E. coli* resistant to at least one antibiotic from three different commonly used classes [20]. *E. coli* are frequently responsible for human urinary tract infections according to the literature. In a study conducted in pediatrics by Savadogo et al. [21] had reported a predominance of *E. coli* in urinary tract infections, this predominance of *E. coli* in urinary tract infections has also been reported by other authors [22, 23]. Similarly, a study conducted over a six-month period by Iqbal et al. [24] showed that the rate of *E. coli* urinary infection was high among *Enterobacteriaceae* strains and represented 63.0%, also at the university hospital of Nepal, a study gives the rate of *E. coli* uropathogenic infection at 62.1% [25]. Table 1 shows the resistance profile of the strains. A high resistance rate was found in this study. Thus, of the fifty-three strains of *E. coli* tested to amoxicillin-clavulanic acid, total resistance 62.26% (*n* = 33) and intermediate resistance 7.54% (*n* = 4) were reported. Resistance to this antibiotic is linked to the production of a beta-lactamase type enzyme. High frequencies of resistance were also observed with trimethoprim-sulfamethoxazole 84.90% (*n* = 45), ciprofloxacin 69.80% (*n* = 37). The resistance rates obtained with trimethoprim-sulfamethoxazole and ciprofloxacin are higher than that obtained by Ramirez-Castillo et *al*., 2018 which was 72.7% [26] and Similar to Ali et al. [27] for trimethoprim-sulfamethoxazole (82%). A total resistance of 7.5% (*n* = 4) and a partial resistance of 13.21% (*n* = 7) have been reported with imipenem. Simultaneous resistance to imipenem and meropenem was observed in strain *E. coli 1473*. Another strain *E. coli 1448* had shown an intermediate result to imipenem and resistance to meropenem. These results show the multi-resistant nature of the strains studied (Table 1). According to the results of the study, fosfomycin appears to be the therapeutic alternative to infections caused by these strains.

**Table 1:** Susceptibility of *Escherichia coli* strains to antibiotics

Species	Antibiotics susceptibility
*E. coli 471*	R ^*NOR, CN, SXT, CIP, CRO, LEV, CFM, F, AMC*^; S ^*FOS*^
*E. coli 919*	R ^*NOR, CN, SXT, CIP, CRO, LEV, CFM, CXM, IMI, AMC*^; S ^*C, F*^
*E. coli P80*	R ^*CN, SXT, CIP, CRO, CFM, CXM, IMI, TCC, AMC*^; S ^*AK*^
*E. coli 1318*	R ^*SXT, CRO, CFM, IMI, TCC, CAZ, AMC*^; S ^*NOR, CN, AK*^
*E. coli 1448*	R ^*NOR, CN, SXT, CIP, CRO, CFM, MEM, FEP, AMC*^; I ^*IMI, AK, TCC*^; S ^*FOS, C, F*^
*E. coli 1473*	R ^*SXT, CIP, IMI, MEM*,*AMC*^; S ^*FOS, CN, CRO, CFM, AK, FEP*^
*E. coli 1476*	I ^*IMI*^; S ^*FOS; CIP, CRO,CFM, F, AMC*^
*E. coli 398*	R ^*NOR, CN, SXT, CIP, CRO, CFM, TC, AMC*^; I ^*IMI*^; S ^*FOS*^
*E. coli 403*	R ^*SXT, TC*^; I ^*F*^; S ^*CN, C, IMI, TCC, AMC*^
*E. coli 405*	R ^*NOR, SXT, CIP, CRO, CFM, TCC, TC, AMC*^; S ^*FOS, CN, C, IMI, F*^
*E. coli 427*	R ^*NOR, SXT, CIP, CRO, CFM, AMC*^; S ^*FOS, CN*^
*E. coli 462*	R ^*NOR, CN, SXT, CRO, LEV, CFM, TCC, TC, NET*^; S ^*FOS, C, IMI*^
*E. coli 476*	R ^*NOR, SXT, CIP, CRO, LEV, AMC, CFM*^; I ^*TCC*^; S ^*FOS, CN, F*,^
*E. coli 478*	R ^*NOR, SXT, CIP, CRO, LEV, CFM, AMC*^; S ^*FOS, CN*^
*E. coli 513*	R ^*CN, SXT, CRO, CFM, TC, AMC*^; I ^*TCC*^; S ^*FOS IMI F*^
*E. coli 514*	R ^*FOS, CRO, CFM, F, AMC*^; S ^*IMI*^
*E. coli 511*	R ^*NOR, SXT, CIP, CRO, CFM, TCC, TC, NET, F, AMC*^; S ^*FOS, CN, IMI*^
*E. coli 583*	R ^*NOR, CN, SXT, CIP, CRO, CFM, C, CAZ, TC, AMC*^; I ^*TCC*^; S ^*FOS, IMI, F*^
*E. coli 596*	R ^*NOR, CN, SXT, CIP, CRO, CFM, CAZ, TC, AMC*^; I ^*TCC*^; S ^*FOS, IMI, F*^
*E. coli 655*	R ^*NOR, CN, SXT, CIP TC, NET, AMC*^; I ^*TCC*^; S ^*FOS, CRO, CFM, IMI, F*^
*E. coli 667*	R ^*NOR, CN, SXT, CRO, CFM, F, AMC*^; S ^*FOS, IMI*^
*E. coli 687*	R ^*NOR, SXT, CIP, CRO, CFM*^*; *S ^*FOS,CN, IMI, TCC, F, AMC*^
*E. coli 678*	R ^*NOR, CN, SXT, CIP, CRO,CFM, TCC, AMC*^; S ^*FOS, IMI, F*^
*E. coli 711*	R ^*NOR, SXT, CIP, CRO, CFM*^; S ^*FOS,CN, IMI, TCC, F, AMC*^
*E. coli 740*	R ^*SXT, CRO, CFM*^; S ^*FOS,NOR, CN, CIP, IMI, NET, F, AMC*^
*E. coli 885*	R ^*SXT*^; S ^*NOR, CN, CIP,CRO, CFM, TCC, AMC*^
*E. coli 874*	R ^*NOR, SXT, CIP, CRO, CFM*^*; S *^*FOS,CN, TCC, F, AMC*^
*E. coli 871*	R ^*NOR, CN, SXT, CIP, CRO,CFM*^; I ^*AMC*^; S ^*FOS, IMI*^
*E. coli P46*	R ^*NOR, SXT, CIP, CRO,CFM, CXM, AMC*^; S^*CN, C, IMI*^
*E. coli P49*	R ^*NOR, CN, SXT, CIP, CRO,CFM, CXM, AMC*^; S ^*C,*^
*E. coli 916*	R ^*SXT*^; I ^*AMC*^; S ^*FOS,NOR, CN, CIP, CRO, LEV, CXM, NET, F,*^
*E. coli 957*	R ^*NOR, SXT, CIP, CRO, LEV CFM AMC*^; S ^*FOS, CN, IMI,AK, F*^
*E. coli 971*	R ^*NOR, CIP, CRO, CFM*^; S ^*FOS CN, SXT, IMI, TCC, F, AMC*^
*E. coli 1060*	R ^*NOR, CIP, CRO, CFM, FEP*^; S ^*FOS CN, C, AK, TCC, F, AMC*^
*E. coli 1081*	R ^*CIP, CRO, LEV, AMC*^; S ^*FOS, SXT, IMI, F*^
*E. coli 1083*	R ^*SXT, CIP, LEV, AMC*^; S ^*FOS, CN,CRO, CFM, IMI, AK, F*^
*E. coli 1084*	R ^*NOR, SXT, CIP*^; I ^*CN*^; S ^*FOS,CRO, F, AMC*^
*E. coli 1099*	R ^*SXT, C, TCC*^; S ^*NOR, CN, CIP, CRO, CFM, IMI, AK, F, AMC*^
*E. coli 1185*	R ^*NOR, SXT, CIP, CRO, CFM, TCC, FEP, AMC*^; I ^*IMI*^*; S *^*FOS CN, F*^
*E. coli 1167*	R ^*SXT, CIP, TCC*^; S ^*CN, CRO, CFM, IMI, AMC*^
*E. coli 1179*	R ^*NOR, SXT, CIP, AK, TCC, AMC*^; I ^*CN, IMI*^; S ^*CRO, CFM, ATM*^^*, F*^
*E. coli P66*	R ^*NOR, CN, SXT, CIP, CRO,AK, ATM*^^*, AMC*^
*E. coli 1220*	R ^*NOR, SXT, CIP, CRO, TCC, ATM*^; I ^*AK*^; S ^*CN, IMI, F, AMC*^
*E. coli 1290*	R ^*CN, SXT, CRO,CFM, CXM, TCC, ATM*^^*, AMC*^; I ^*IMI*^; S ^*C, F*^
*E. coli 1291*	R ^*NOR, CN, SXT, CIP, CRO,CFM, C, AK, TCC, AMC*^; S ^*IMI, F*^
*E. coli 1321*	R ^*NOR, SXT, TCC, AMC*^; I ^*IMI, AK*^; S ^*CRO, CFM, CAZ*^
*E. coli 1283*	R ^*C, TCC, AMC*^; S ^*NOR, CN, CIP, CRO, CFM, CXM, F*^
*E. coli 1701*	R ^*NOR, CN, CIP, CRO, CFM, CXM, TCC, AMC*^; S ^*FOS, C,IMI, AK, F*^
*E. coli 1830*	R ^*NOR, CN SXT, TCC, AMC*^; S ^*FOS, C,IMI, AK, F*^
*E. coli 1831*	R ^*NOR, CN,SXT CIP, CRO, TCC*^; S ^*FOS, C,IMI, AK, F*^
*E. coli 469*	R ^*CIP, LEV, C, TCC, CAZ, ATM*^; S ^*FOS, CN, NET*^
*E. coli 543*	R ^*NOR, CN, SXT, CIP, CRO, CFM, C, AMC*^; I ^*TCC*^; S ^*FOS, IMI*,^
*E. coli 669*	R ^*NOR, CN, SXT, CIP, CRO, CFM*^*;* S ^*FOS,IMI, F, AMC*,^

Legend: *R* Resistant, *I* Intermediary, *S* Sensitive, *FOS* Fosfomycin, *NOR* Norfloxacin, *CN* Gentamicin, *SXT* Triméthoprim + Sulfaméthoxazole, *CIP* Ciprofloxacin, *CRO* Ceftriaxon, *LEV* Levofloxacin, *CEM* Cefixim, *C* Chloramphénicol, *CXM* Cefuroxim, *IMI* Imipenem, *AK* Amikancin, *TCC* Ticarcillin-clavulanat, *CAZ* Ceftazidim, *MEM* Meropenem *FEP* Cefepim, *TC* Ticarcillin, *ATM* Aztreonam, *NET* Netilmicin, *F* Nitrofuran, *AMC* Amoxicilline-Acide clavulanique

### Carbapenemase production

**Fig. 1:**
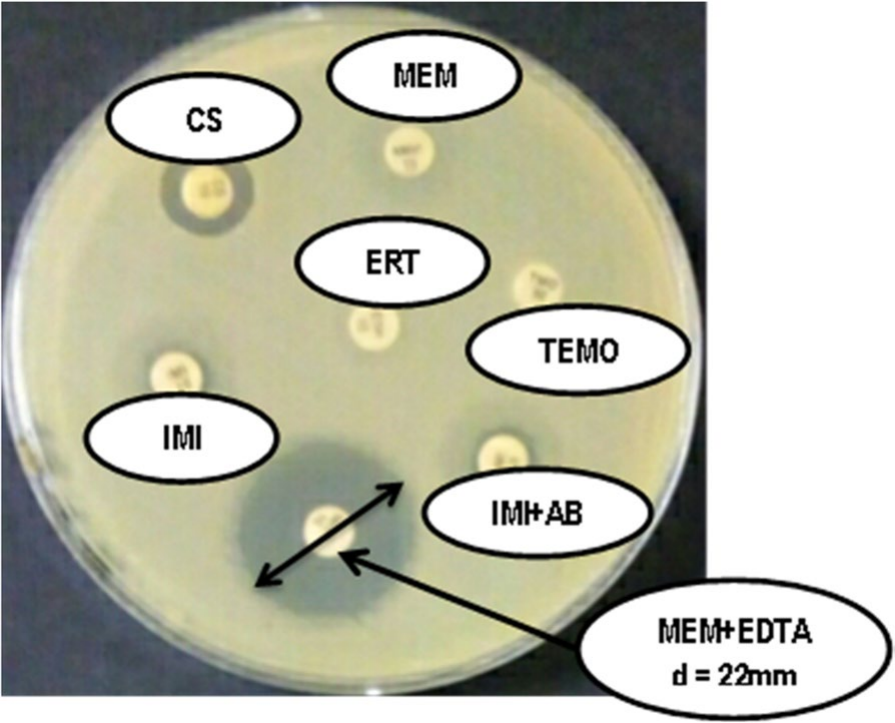
Antibiogram of *E. coli 1318*. Legend: CS: Colistin, MEM: Meropenem, ERT: Ertapenem, IMI: Imipenem, TEMO: Temocillin, IMI + AB: Imipenem + Boric Acid, MEM + EDTA: Meropenem + Ethylene Diamin Tetraacetic Acid

Carbapenemase production was detected in a single multidrug-resistant strain, *E. coli 1318*. Figure 1 shows metallo-beta-lactamase production of *E. coli 1318*. Inhibition of bacterial growth was observed around the meropenem disc in the presence of EDTA. While no growth was observed around the meropenem disc alone. Similarly, an absence of inhibition was observed around the disk of imipenem + boric acid and imipenem alone. Franklin et al*.* had highlighted by the phenotypic method strains of *Enterobacteriaceae* producing carbapenemase including *E. coli* [28].

### Molecular identification by (GTG)_5_-PCR

**Fig. 2:**
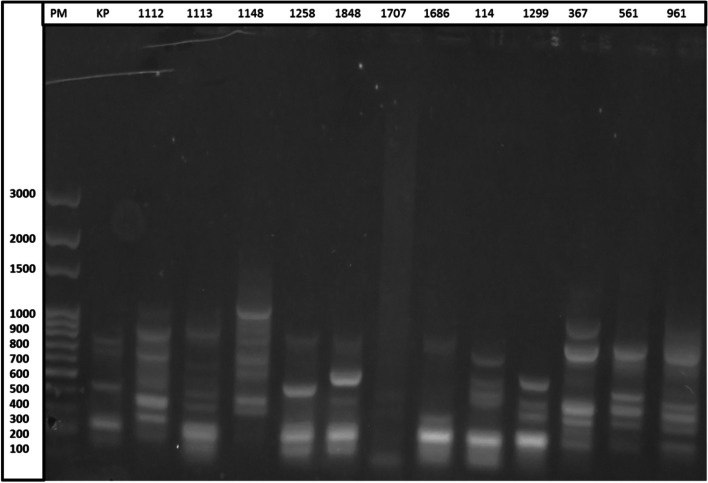
Profile of fingerprinting of strains with *Klebsiella pneumoniae ATCC 700603*. Legend: PM: molecular weight marker, KP: *Klebsiella pneumoniae ATCC 700603*, *1112 à 961: strains identity*

All strains of *E. coli* exhibited a (GTG)_5_-PCR profile. Figures 1 and 2 shows the profile genetic fingerprints of the strains studied. In the bacterial genome, certain genetic sequences are repeated in several places. The rep-PCR primers are made to amplify the sequences around the repeating elements. Mutations can change the genome sequence and rep-PCR can be used as a diagnostic tool. Indeed, while rep-PCR DNA fingerprinting patterns are considered stable over many generations of microbial growth [29]. Rep-PCR fingerprinting can be used to study microbial genome evolution [9]. The rep-PCR made it possible to study fifty-three strains of *E. coli*. The electrophoresis of the PCR products enabled us to obtain profiles of the genetic fingerprints of each strain (Figs. 1 and 2). These profiles reveal bands characteristic of the reference strains used in this study; *Klebsiella pneumoniae ATCC 700603* (five characteristic bands at 100 bp, 200 bp, 400 bp, 700 bp and 800 bp in Fig. 1), as well as *E. coli* ATCC 25,922 (four characteristic bands at 200 bp, 300 bp, 500 bp and 1000 bp in Fig. 2) and *Pseudomonas aeruginosa ATCC 27853* (five characteristic bands at 100 bp, 200 bp, 300 bp, 500 bp and 1000 bp Fig. 2). Twenty-two groups were obtained by analyzing the genetic profiles generated by rep-PCR (Figs. 1 and 2). The number of characteristic bands of *E. coli* varied from 2 to 6 and the group having the characteristic bands at 200 bp, 300 bp, 400 bp and 500 bp was the most representative and corresponding to 15% of *E. coli* species. Overall, the fingerprint profiles generated by the studied strains were different from those reported by Khare et al. on strains of *E. coli* with a number of bands varying from 15 to 24 [8]. This variability can be attributed to environmental conditions and the genetic evolution of the strains [30, 31]. 75.47% of the *E. coli* strains studied showed four to five characteristic bands, 45.28% showed four characteristic bands like the reference strain *E. coli ATCC 25922* but differed only in their position. Some studies have shown that *E. coli* fingerprints obtained from the same animal are similar, but not always identical [31]. A small proportion of the strains show two characteristic bands and represents 3.77%.

### Phylogenetic affiliation

**Fig. 3:**
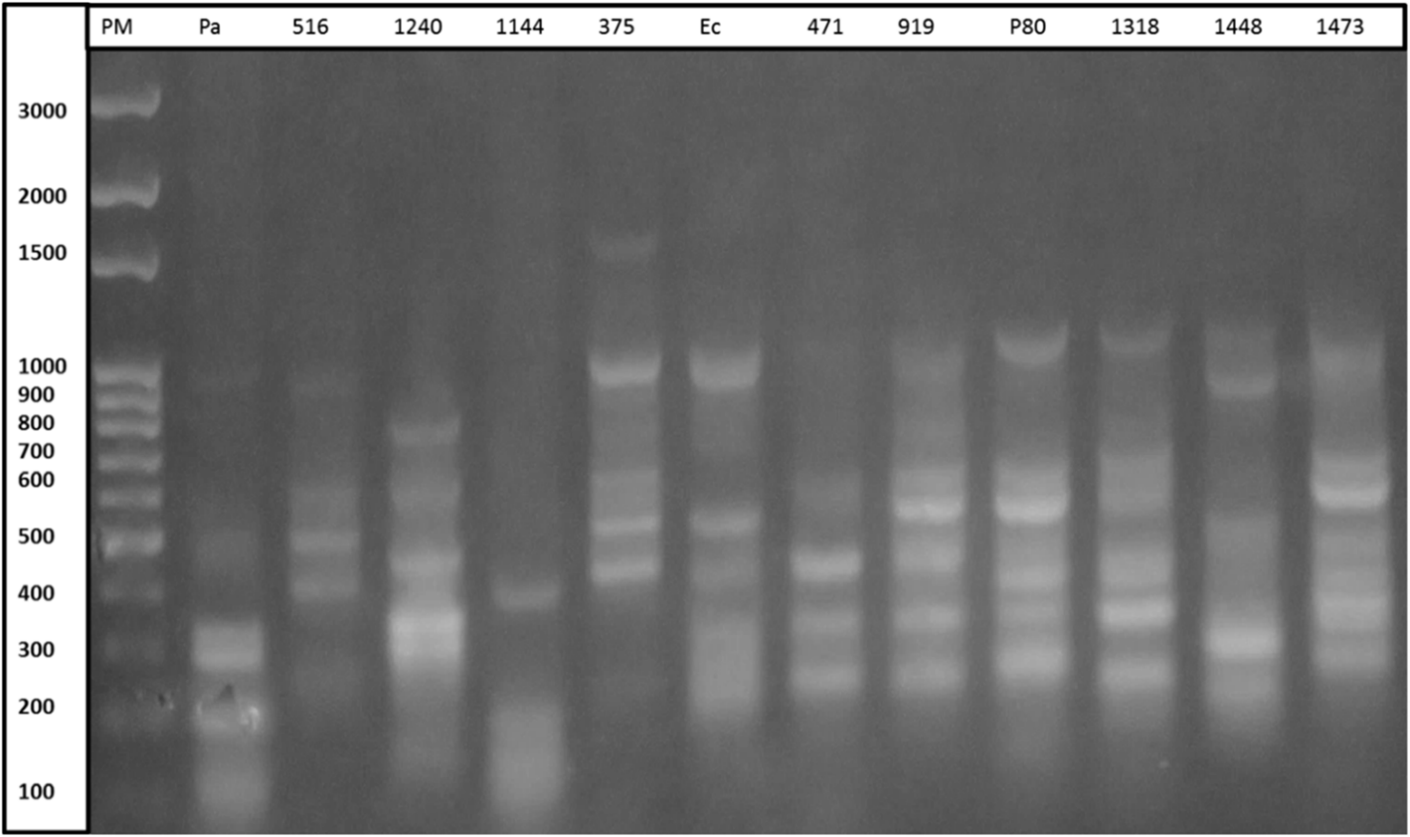
Profile of fingerprinting of strains with *E. coli* ATCC *25922* and *Pseudomonas aeruginosa ATCC 27853*. Legend: PM: molecular weight marker, Ec: *E. coli ATCC 25922, Pa: Pseudomonas aeruginosa ATCC 25053.* 516, 1240, 1144, 375, 471, 919, P80, 1318, 1448, 1473: strains identity

**Fig. 4:**
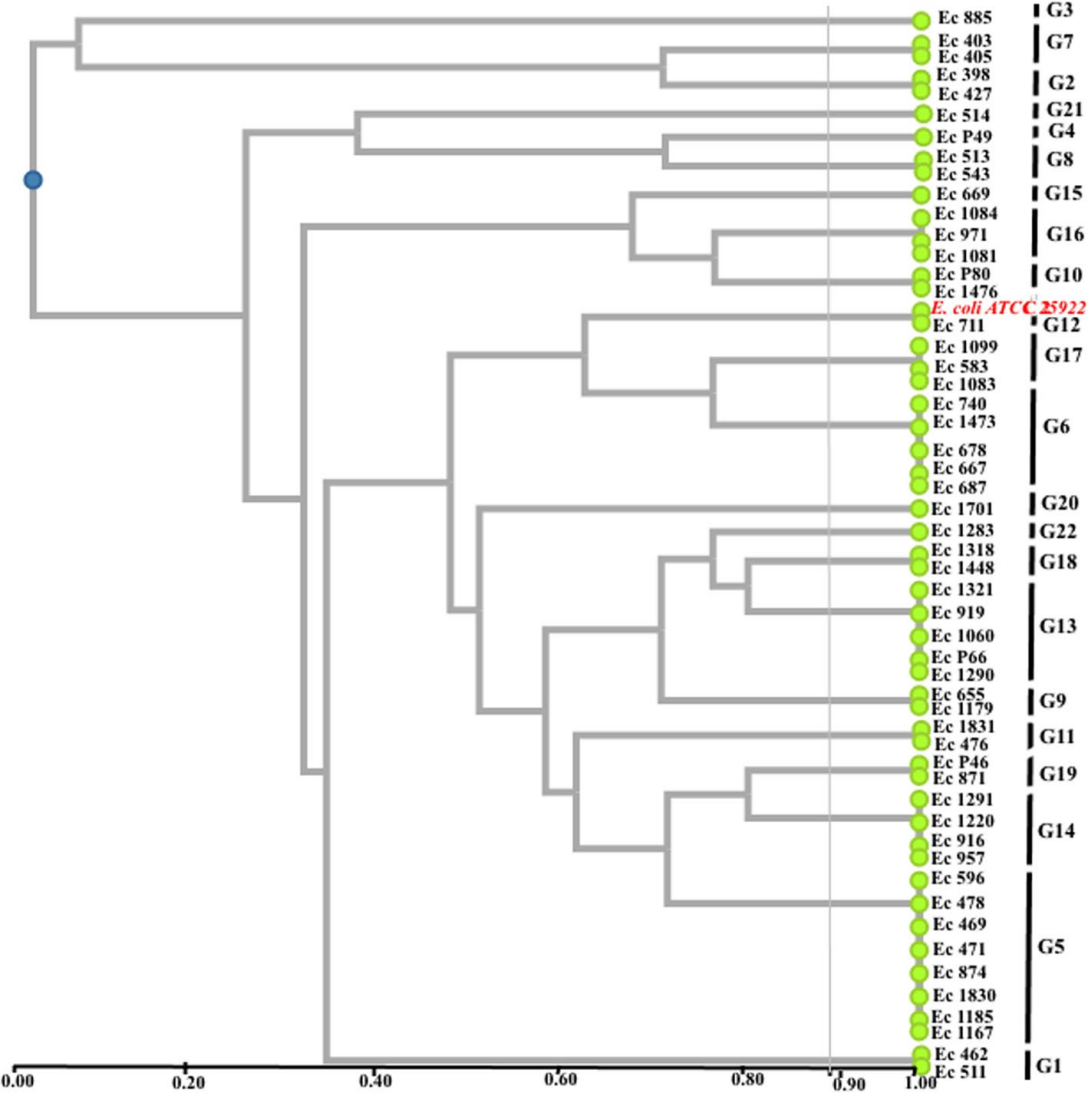
Dendrogram based on (GTG)_5_-PCR fingerprints of *E. coli* clinical strains according UPGMA algorithm

The phylogenetic tree obtained presents the relationship between the different strains studied thanks to their genetic profile in comparison with the reference strain *E. coli ATCC 25922*. The family relationship of the strains is determined by the vertical straight line at 88% on the dendrogram. Through the dendrogram, it can be seen that the reference strain *E. coli ATCC 25922* is positioned in the same place as the strain *E. coli 711*. Analysis of the phylogenetic tree shows that there is diversity between the strains studied. Thus twenty-two groups were obtained. This shows a weak phylogenetic relationship between the strains studied (similarity rate less than 88%, Fig. 3). khare et *al*. and Mohapatra et al. had used the same method to grouping *E. coli* strains [8, 32], similarly Ranjbar and. Afshar had grouped together the strains of uropathogenic *Klebsiella pneumoniae* by the same method [18]. According to molecular analysis and phylogeny tree based profile of fingerprinting of strains, we find that the strains of groups G13 (P66), G10 (P80) and G19 (P46) isolated from pus could also be the cause of urinary tract infections due to of their affiliation at strains responsible of urinary pathologies according to Fig. 4. These strains have no particularity as regards of pathologies caused. However, the group G4 (P49) strain isolated from pus is unique from other urinary tract infection strains, so it might be specific only for suppurative infections. However, the strains of certain groups G1, G2, G3, G5, G6, G7, G8, G9, G11, G12, G14, G15, G16, G17, G18, G20, G21 and G22 are only uropathogens according to their affiliation (Fig. 4). Indeed, some authors had used GTG_5_ method to discriminate the strains of *E. coli* responsible for human and animal pathologies [8, 33, 34].

## Conclusion

This study allowed us to assess antibiotic susceptibility and identify strains of *E. coli* isolated from pathological products. Multiresistant strains of the MDR type were identified in this study. Some strains have shown resistance to carbapenems. (GTG)_5_-PCR allowed molecular identification and grouping of these strains into subgroups with common characteristics.

## Supplementary Information

**Additional file 1.**

## Data Availability

Yes.
