# Supplementary material for: (GTG)5-PCR fingerprinting of multi-drug resistant Escherichia coli bacteria isolates from hospital in Ouagadougou, Burkina Faso
Source: BMC Microbiol. 2022 Apr 29;22:118. doi: 10.1186/s12866-022-02537-7 (PMC9052641; doi:10.1186/s12866-022-02537-7)
Supplement: Supplementary file 1 — Additional file 1. [file 12866_2022_2537_MOESM1_ESM.pdf]

Supplementary material

The analysis carried out in this study consisted in making a discrimination of the strains with the GTG5 primer. This discrimination consists in seeking the characteristic bands of each strain. The construction of the phylogenetic tree is based on the presence (denoted 1) or the absence (denoted 0) in the profile of the genetic fingerprint of the strains within the repeated regions of the bacterial genome. Thus obtaining the phylogenetic tree with the software (http://genomes.urv.cat/UPGMA/index.php?entrada=Example2) does not require sequencing or depositing the sequence in a database.

Procedure for obtaining the phylogenetic tree

We take the data contained in the DATA file, then we deposit it in the D-UPGMA input space and finally click on the submit button to generate the tree.

The paint software was used to modify the color, the entry of the strain codes and the groups.

The following links were used to generate the phylogenetic tree:

http://genomes.urv.cat/UPGMA/index.php?entrada=Example2

http://genomes.urv.cat/UPGMA/UPGMAboot_v12.cgi

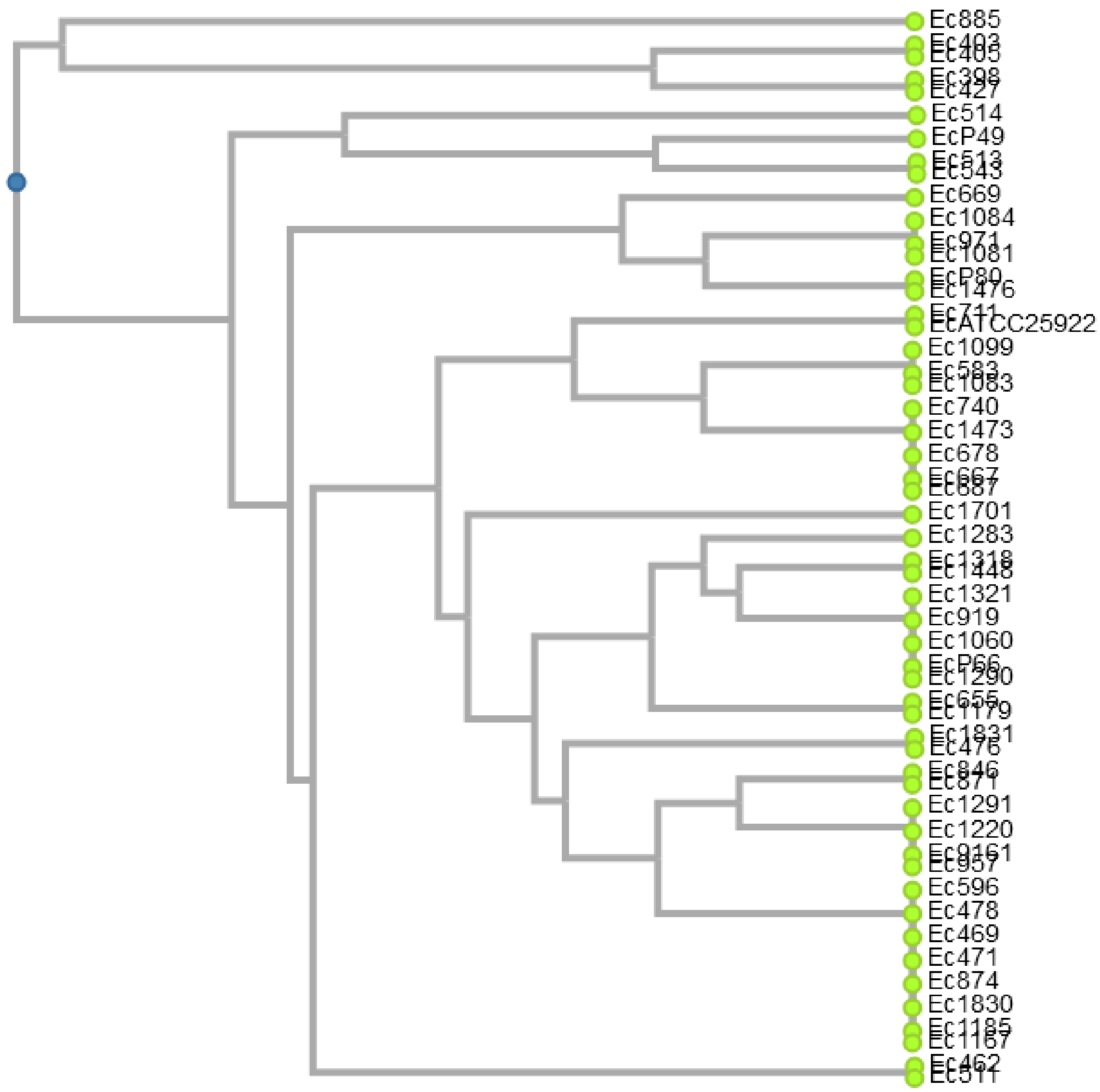

Ec885
Ec403
Ec405
Ec398
Ec427
Ec514
EcP49
Ec513
Ec543
Ec669
Ec1084
Ec971
Ec1081
EcP80
Ec1476
Ec711
EcATCC25922
Ec1099
Ec583
Ec1083
Ec740
Ec1473
Ec678
Ec667
Ec687
Ec1701
Ec1283
Ec1318
Ec1448
Ec1321
Ec919
Ec1060
EcP66
Ec1290
Ec655
Ec1179
Ec1831
Ec476
Ec846
Ec871
Ec1291
Ec1220
Ec9161
Ec957
Ec596
Ec478
Ec469
Ec471
Ec874
Ec1830
Ec1185
Ec1167
Ec462
Ec511

DATA

>Ec462

0 1 0 0 0 0 1 0 0 0 0 0 0 0 0

>Ec511

0 1 0 0 0 0 1 0 0 0 0 0 0 0 0

>Ec398

0 0 1 0 0 0 1 0 1 0 0 0 0 0 0

>Ec427

0 0 1 0 0 0 1 0 1 0 0 0 0 0 0

>Ec885

0 0 1 0 0 0 0 1 0 0 0 1 0 0 0

>EcP49

0 1 0 0 0 1 0 0 0 0 0 1 0 0 0

>Ec1185

0 1 0 1 0 1 1 0 0 0 0 0 0 0 0

>Ec1167

0 1 0 1 0 1 1 0 0 0 0 0 0 0 0

>Ec1830

0 1 0 1 0 1 1 0 0 0 0 0 0 0 0

>Ec874

0 1 0 1 0 1 1 0 0 0 0 0 0 0 0

>Ec471

0 1 0 1 0 1 1 0 0 0 0 0 0 0 0

>Ec469

0 1 0 1 0 1 1 0 0 0 0 0 0 0 0

>Ec478

0 1 0 1 0 1 1 0 0 0 0 0 0 0 0

>Ec596

0 1 0 1 0 1 1 0 0 0 0 0 0 0 0

>Ec667

0 1 0 1 0 0 1 0 0 0 1 0 0 0 0

>Ec687

0 1 0 1 0 0 1 0 0 0 1 0 0 0 0

>Ec678

0 1 0 1 0 0 1 0 0 0 1 0 0 0 0

>Ec1473

0 1 0 1 0 0 1 0 0 0 1 0 0 0 0

>Ec740

0 1 0 1 0 0 1 0 0 0 1 0 0 0 0

>Ec403

0 0 1 0 1 0 1 0 1 0 0 0 0 0 0

>Ec405

0 0 1 0 1 0 1 0 1 0 0 0 0 0 0

>Ec513

0 1 0 1 0 1 0 0 0 0 0 1 0 0 0

>Ec543

0 1 0 1 0 1 0 0 0 0 0 1 0 0 0

>Ec655

0 1 0 0 0 1 1 0 1 0 0 0 0 0 0

>Ec1179

0 1 0 0 0 1 1 0 1 0 0 0 0 0 0

>EcP80

0 0 0 1 0 1 0 0 1 0 0 0 1 0 0

>Ec1476

0 0 0 1 0 1 0 0 1 0 0 0 1 0 0

>Ec1831

0 0 0 1 0 1 1 0 0 0 1 0 0 0 0

>Ec476

0 0 0 1 0 1 1 0 0 0 1 0 0 0 0

>Ec711

0 1 0 1 0 0 1 0 0 0 0 0 1 0 0

>EcP66

0 1 0 1 0 1 1 0 1 0 0 0 0 0 0

>Ec1290

0 1 0 1 0 1 1 0 1 0 0 0 0 0 0

>Ec1060

0 1 0 1 0 1 1 0 1 0 0 0 0 0 0

>Ec919

0 1 0 1 0 1 1 0 1 0 0 0 0 0 0

>Ec1321

0 1 0 1 0 1 1 0 1 0 0 0 0 0 0

>Ec916-1

0 1 0 1 0 1 1 0 0 0 1 0 0 0 0

>Ec957

0 1 0 1 0 1 1 0 0 0 1 0 0 0 0

>Ec1220

0 1 0 1 0 1 1 0 0 0 1 0 0 0 0

>Ec1291

0 1 0 1 0 1 1 0 0 0 1 0 0 0 0

>Ec669

0 1 0 0 0 1 0 0 1 0 0 0 1 0 0

>Ec971

0 1 0 1 0 1 0 0 1 0 0 0 1 0 0

>Ec1081

0 1 0 1 0 1 0 0 1 0 0 0 1 0 0

>Ec1084

0 1 0 1 0 1 0 0 1 0 0 0 1 0 0

>Ec583

0 1 0 1 0 0 1 0 0 0 1 0 1 0 0

>Ec1083

0 1 0 1 0 0 1 0 0 0 1 0 1 0 0

>Ec1099

0 1 0 1 0 0 1 0 0 0 1 0 1 0 0

>Ec1318

0 1 0 1 0 1 1 0 1 0 0 0 1 0 0

>Ec1448

0 1 0 1 0 1 1 0 1 0 0 0 1 0 0

>Ec846

0 1 0 1 0 1 1 0 0 0 1 0 1 0 0

>Ec871

0 1 0 1 0 1 1 0 0 0 1 0 1 0 0

>Ec1701

1 1 0 1 0 1 1 0 0 1 0 0 0 0 0

>Ec514

0 1 0 0 0 1 0 0 0 0 1 1 0 1 1

>Ec1283

0 1 0 1 0 1 1 0 1 1 0 0 0 0 0

>EcATCC25922

0	1	0	1	0	0	1	0	0	0	0	0	1	0	0
